# Antifungal Effect of Oregano Essential Oil Against *Penicillium expansum* on *Pyrus sinkiangensis*

**DOI:** 10.3390/jof10110752

**Published:** 2024-10-30

**Authors:** Qun Liu, Li Li, Zhenyuan Yang, Xiaodi Xiong, Qi Song, Baishu Li, Hang Zou, Lixiang Zhang, Tao Liu

**Affiliations:** 1 Chinese Academy of Inspection and Quarantine, No. A3, Gaobeidian North Road, Chaoyang District, Beijing 100123, China; liuq@caiq.org.cn (Q.L.);; 2 Technology Innovation Center of Animal and Plant Product Quality, Safety and Control, State Administration for Market Regulation, Beijing 100176, China; 3 Liuzhou Quality Inspection and Testing Research Center, Liuzhou 545001, China; 4 College of Advanced Agriculture and Ecological Environment, Hei Longjiang University, No. 74, Xuefu Road, Nangang District, Harbin 150080, China

**Keywords:** *Penicillium expansum*, oregano essential oil, pears, antifungal activity, quality attributes, postharvest management

## Abstract

Given the increasing demand for fruit safety assurance and environmental protection, plant essential oils have gained significant attention as natural alternatives for control of postharvest decay caused by various pathogens. In postharvest management, it is particularly important to effectively control postharvest decay without compromising the quality attributes of fruits. Although oregano essential oil (OEO) has been extensively studied for its antimicrobial properties against various postharvest pathogens, few studies have focused on its interactions with postharvest fruits. In this study, OEO was applied for management of postharvest decay of *Pyrus sinkiangensis* caused by *Penicillium expansum*, and its antifungal mechanisms and impacts on the quality attributes of pears were investigated. The OEO exhibited notable inhibitory effects, with determined MIC (0.02%) and MFC (0.04%) against *P. expansum*, which highlighted its potential as a viable alternative to synthetic fungicides. Our findings revealed that OEO disrupted *P. expansum* by compromising the integrity of the fungal plasma membrane, as evidenced by changes in plasma membrane permeability and the leakage of cellular components. The OEO treatment significantly reduced weight loss, maintained firmness, and preserved soluble-solid content in the treated pears. In addition, OEO treatment stimulated the intrinsic antioxidant mechanisms of pears, as demonstrated by elevated activities of superoxide dismutase and catalase during storage. This study provides compelling evidence for the antifungal and quality-preserving properties of OEO in the postharvest management of pears, underscoring its potential as an alternative to synthetic fungicides for controlling postharvest decay. The elucidation of the interaction between OEO and *P. sinkiangensis* would greatly enhance our comprehensive understanding of the biological activities of OEO and facilitate its practical application in the postharvest management of pears.

## 1. Introduction

Fruits play a pivotal role in human nutrition by providing essential vitamins, carbohydrates, and trace elements, thereby constituting a crucial component of the daily diet [1]. However, the deterioration of fruits due to microbial contamination during production, storage, and distribution presents significant economic and health challenges [2]. Among the various pathogens, *Penicillium expansum* is particularly notorious for causing blue mold in apples, peaches, and pears, which not only leads to substantial economic losses but also poses health risks due to mycotoxin exposure [3]. While the application of synthetic fungicides has proven effective in managing postharvest decay, it raises serious concerns regarding environmental impact, potential human health risks, and the development of resistance in fungal populations [4]. Consequently, there has been a growing interest in exploring safer and more sustainable alternatives to traditional chemical treatments [5,6].

Among the various natural alternatives, plant essential oils have emerged as a promising option due to their natural origin, biodegradability, and broad-spectrum antimicrobial properties [7]. Essential oils, derived from a diverse array of plant materials, are particularly recognized for their complex mixtures of bioactive components, including terpenes, phenylpropanoids, and sulfur-containing compounds [8,9]. These bioactive components confer the broad-spectrum antimicrobial effects upon the essential oils, effects which are instrumental in mitigating the development of the resistance commonly associated with single-target synthetic preservatives [10,11]. Among these essential oils, oregano essential oil (OEO), which predominantly contains carvacrol and thymol, has demonstrated significant antifungal properties against a variety of pathogens [12].

Fincheira et al. demonstrated that OEO exhibited significant inhibitory effects on the growth and spore germination of *P. expansum*, thereby highlighting its potential as a natural preservative [13]. Further investigations have indicated that OEO not only reduced fungal load but also extended the shelf lives of fruits, such as apples, that were susceptible to *P. expansum* [14]. The antifungal mechanisms employed by OEO are multifaceted, encompassing the disruption of fungal cell membrane integrity, interference with enzymatic activities, and the induction of oxidative stress within fungal cells [15]. As the primary monoterpenoid phenols found in OEO, carvacrol and thymol have garnered significant attention and been widely studied due to their potent antimicrobial properties, which are effective against a broad spectrum of microorganisms. Carvacrol (C_10_H_14_O) and thymol (C_10_H_14_O) are structurally similar monoterpenic phenols but differ slightly in the position of the hydroxyl group attached to the aromatic ring, contributing to their similar yet distinct biological activities [16]. Wijesundara et al. [17] revealed that carvacrol exhibited instantaneous bactericidal activity against *Streptococcus pyogenes* through compromising the cell membrane integrity, as evidenced by the leakage of cytoplasmic content. Thymol was reported to inhibit the growth of *Escherichia coli* by inducing the permeabilization and depolarization of the cytoplasmic membrane [18]. In addition, carvacrol was found to show a synergistic effect in combination with thymol against *Micrococcus luteus*, with a FIC*_I_* value of 1.03 [19]. A thorough comprehension of these mechanisms is essential for enhancing the practical application of OEO, particularly in the formulation of effective postharvest management strategies for fruits.

In addition to its well-documented antifungal properties, OEO has garnered considerable attention for its influence on the quality attributes of treated fruits, including texture, flavor, color, and nutritional content [20]. The application of OEO on strawberries has been shown to significantly enhance sensory qualities, thereby improving flavor and extending shelf life without compromising nutritional value [21]. In addition, when OEO was utilized in combination with advanced technologies such as modified atmosphere packaging, the retention of critical sensory and nutritional properties in fruits were optimized, further prolonging shelf life without compromising taste [22,23]. Therefore, a comprehensive evaluation of both the antifungal efficacy and the impact on fruit quality is essential for the successful application of OEO in fruit preservation.

Despite the documented antifungal properties of OEO, there remains a significant gap in understanding its specific mechanisms against *P. expansum*, as well as its impact on the quality attributes of fruits. The present study aims to investigate the specific antifungal mechanisms of OEO against *P. expansum* and to assess its effects on the quality and physiological attributes of Kuerle pears, a variety esteemed for its commercial and nutritional significance. This research will integrate microbiological, biochemical, and sensory analyses to offer a comprehensive evaluation of the use of OEO as a sustainable approach used to manage fungal decay in postharvest fruits. By elucidating these mechanisms and their practical implications, this study seeks to contribute valuable insights into the development of natural preservation strategies that not only ensure safety and extend shelf life, but also enhance the overall quality of the fruits.

## 2. Materials and Methods

### 2.1. Materials and Chemicals

Oregano essential oil with 98% purity, ergosterol, Tween 80, and propidium iodide were purchased from Yuanye Bio-Technology Co., Ltd. (Shanghai, China). Potato Dextrose Agar (PDA) medium, 90 mm Petri dishes, 40 μm nylon cell filters, lactic acid cotton blue-staining solution, and a live/dead cell activity/cytotoxicity detection kit were sourced from Solaibao Technology Co., Ltd. (Beijing, China). *P. expansum* T01 was obtained from the laboratory of Tian Shiping at the Institute of Botany, Chinese Academy of Sciences (Beijing, China), and was maintained on PDA slants at 4 °C.

### 2.2. Preparation of Spore Suspension

The spore suspensions were prepared based on the techniques described in a previous study, with slight modifications [24]. *P. expansum* was cultured on PDA agar plates at room temperature (25 °C) for three days. Afterward, 3 mL of sterile saline was added to the Petri dish. Hyphae and spores were gently scraped off and filtered through a 40 μm cell filter to collect the spore suspension. Spore counts were conducted using a hemocytometer (Shanghai Qiujing Biochemical Reagent Instrument Co., Ltd., Shanghai, China) under a microscope. Spore concentrations were adjusted to the required levels through gradient dilution and stored at 4 °C.

### 2.3. Validation of Penicillium expansum

#### 2.3.1. Sequencing of Internal Transcribed Spacer (ITS)

Hyphae were lysed using a lysis kit (Takara Biomedical Technology Co., Ltd., Beijing, China), and the supernatant served as the template for ITS fragment amplification. Primers ITS1-F (5′-TCCGTAGGTGAACCTGCGG-3′) and ITS4-R (5′-TCCTCCGCTTATTGATATGC-3′) were employed, producing DNA fragments approximately 500–700 base pairs long. PCR products were sequenced by Beijing Ruibo Biotech Co., Ltd. (Beijing, China). and analyzed via BLAST against the NCBI database.

#### 2.3.2. Morphological Observation and Pathogenicity

The morphology of spores and mycelium was examined under a ZEISS Imager A1 microscope. Colonial morphology was documented photographically after 5–7 days of culture on PDA plates.

The pathogenicity of *P. expansum* on *Pyrus sinkiangensis* pears was assessed by inoculating 10 μL of spore suspension (1 × 10^6^ CFU/mL) into wounds on the pears, following surface sterilization and drying. The surface of *Pyrus sinkiangensis* was sterilized by soaking in 0.01% sodium hypochlorite solution for 10 min. One wound (2 mm deep and 2 mm wide) was made on the equators of the pears with a sterile loop. Control fruits received sterile water inoculations. The diameter of rot tissue was measured daily over one week at room temperature (25 °C).

### 2.4. Antifungal Effect Evaluation

#### 2.4.1. Determination of Minimum Inhibitory and Fungicidal Concentrations (MIC and MFC)

MIC and MFC were determined as per our previous methodology [25]. OEO was dissolved in Tween 80 and added to 5 mL of Potato Dextrose Broth (PDB) medium at various concentrations along with 100 μL of spore suspension (1 × 10^6^ CFU/mL). The cultures were incubated at 28 °C for 48 h. Concentrations showing no fungal growth were recorded as MIC, and further cultured in fresh PDB medium without essential oil for another 48 h to establish MFC. For determination of MIC and MFC, the groups without addition of essential oils were set as controls. Each group contained 3 replicates.

#### 2.4.2. Spore Germination and Mycelium Growth

Spore germination was determined according to the techniques described in a previous study, with slight modifications [24]. Briefly, 100 μL of spore suspension was inoculated into PDB medium containing different concentrations of OEO, and incubated at 28 °C for 96 h. An optical microscope (400× magnification, ZEISS Imager A1) and lactophenol cotton blue staining were utilized for enhanced visibility of the germination process. The spores were regarded as germinated when the germination tube was longer than or equal to the spore diameter. For observation of hyphae expansion, 10 μL of spore suspension (1 × 10^6^ spores/mL) was dropped on the center of PDA plate supplemented with OEO at various concentrations (0, 1/2 MIC, MIC, or 2 MIC), and incubated at 28 °C for 7 days. The agar plate without addition of OEO was used as blank control. The growth of hyphae was recorded by monitoring colony diameter by the cross-crossing method. All of the experiments were repeated three times.

### 2.5. Plasma Membrane Integrity and Permeability

#### 2.5.1. Plasma Membrane Integrity

The effect of OEO on the membrane integrity of *P. expansum* was evaluated by using propidium iodide (PI) stanning, based on the methods described in a previous study [25]. Spore suspensions were incubated with varying concentrations of OEO (0 to 2× MIC) at 28 °C for 12 h. The spores were harvested, washed with PBS, and stained with PI (500×) for 15 min in the dark. The stained spores were then observed under a fluorescence microscope (ZEISS Axio Imager A1, Oberkochen, Germany). Three replications were conducted in each group. For each sample, three field views were chosen randomly and the spore numbers were counted under the bright field. The membrane integrity can be calculated by the following formula: membrane integrity (%) = [1 − (number of stained spores/number of total spores)] × 100.

#### 2.5.2. Extracellular Electric Conductivity

The extracellular conductivity was measured with a conductivity meter, the DDS-307A (INESA, Shanghai, China), referring to a previous study [26]. An amount of 200 μL of spore suspension (1 × 10^7^ spores/mL) was mixed with 2 mL sterile water, and the mixtures were supplemented with OEO to reach final concentrations of 1/2 MIC, MIC, and 2 MIC, respectively. The control group was supplemented with an equal amount of sterile water. The mixtures were incubated at 28 °C, and the extracellular conductivity was determined at intervals of a half-hour or one hour. There were 3 replicates created in each group, and each replicate was measured 3 times.

#### 2.5.3. Protein Leakage

Bradford’s method was used for determining protein content [27]. The hyphae were harvested after a 5-day culture of spores in 40 mL PDB medium and washed twice with PBS. A 0.2 g portion of wet hyphae was mixed with 400 μL mixtures of PBS containing different concentrations of OEO (0, 1/2 MIC, MIC, and 2 MIC). The mixtures were incubated in a shaker for 24 h at 28 °C; this was followed by centrifugation at 10,000× *g* for 10 min to obtain the supernatant. The supernatant was used for protein concentration determination. There were 3 replicates in each group, and each replicate was measured 3 times.

### 2.6. Preparation and Application of Plant Essential Oil Emulsion

The oregano–chitosan emulsion was prepared by referring to the method described by Gutiérrez et al. [28], with modifications. Chitosan was dissolved in 1% glacial acetic acid to reach a final concentration of 1%; this was followed by thorough stirring and ultrasonication for 10 min. OEO emulsified with 1% Tween 80 was then added to the mixture to achieve final concentrations of 0.04% (2 MIC) for the essential oil and 0.1% for Tween 80. The mixtures were further homogenized by full agitation and ultrasonication for an additional 10 min. The preparation of artificially inoculated pears was consistent with the methods described in Section 2.3.2. The artificially contaminated pears were soaked in the prepared emulsion solution for 3 min, while the control group was soaked in water. Lesions were photographed and lesion diameters were recorded daily.

### 2.7. Determination of Quality Attributes

#### 2.7.1. Appearance and Color

The color of pears was measured every three days during a 15-day storage period at room temperature (25 °C). Pears of the same size were used for evaluation, and all experiments were performed with three replicates. The surface color was represented by L* (lightness), a* (redness), and b* (yellowness) [29], measured at six locations near the lesion on each sample using a CR-10 colorimeter according to the description of Liu et al. [30]. The colorimeter was calibrated using a white plate before use, and the values were represented as mean ± standard deviation.

#### 2.7.2. Firmness, Water Loss, Soluble-Solid Content (SSC), and Titratable Acidity (TA)

Fruit hardness was measured using a TMS-PRO texture analyzer (FTC company, Rockland, MA, USA). For firmness determination, three points on the surface of the Korla pear at the equator were selected. The probe (5 mm) penetrated the pears to a depth of 8 mm at a speed of 6 cm/min. The force sensor range was 250 N and the trigger force was 0.05 N. Three points were measured near the lesion, and the average maximum force required during the puncture process was shown as hardness, expressed in Newtons (N).

For water-loss determination, each control and essential oil treatment group consisted of five pears. The weight of the pears was measured daily, and the weight loss rate was calculated and represented as mean ± standard deviation. For SSC and TA determination, 10 g of pear tissue was ground into homogenized juice, and 0.3 mL of juice was used for measurement. The SSC was measured with a handheld sugar meter, Pocket PAL-1 (Aito, Nagoya, Japan), and the TA was determined using an acidity meter, GMK-708 (G-WON Company, Seoul, Korea). All determinations were repeated three times.

#### 2.7.3. Respiratory Intensity

Respiratory intensity was measured every three days during a 15-day storage period at room temperature (25 °C). For each determination, seven fruits were collected and placed into a 6 L glass jar, which was then sealed and stored at room temperature. Samples of 0.4 mL of gas were drawn from the jar at intervals of two hours to determine the respiration rate of the pear fruits. The concentration of CO_2_ was detected using a gas chromatograph, following the method described by Liu et al. [30]. All determinations were repeated three times. The respiration rate was expressed as mL of CO_2_ Kg^−1^ h^−1^.

#### 2.7.4. Determination of Enzyme Activity

The extraction of crude enzyme solution was prepared as follows: Pear samples were collected every three days and cut into small pieces, 10 g of which was ground to powder in liquid nitrogen. The powder was suspended in 90 mL of cold potassium phosphate buffer (100 mM, pH 7.4). The homogenized mixture was centrifuged at 10,000× *g* for 10 min to obtain the crude enzyme extracts, which were then used to determine enzyme activity.

For peroxidase (POD) activity determination, 200 μL of crude extraction solution was added to 3.6 mL of reaction buffer containing 0.1% methoxyphenol, 0.05% hydrogen peroxide, and 100 mM potassium phosphate buffer (pH 7.4). POD activity was measured at room temperature by monitoring the increase in absorbance at 470 nm. For superoxide dismutase (SOD) activity determination, the crude SOD extraction was reacted with tetrazolium blue (NBT) under 4000 × sunlight for 20 min, and the absorption at 560 nm was recorded. One unit of SOD represented the enzyme amount required to inhibit 50% of the NBT photo-reduction reaction. Catalase (CAT) activity was determined by detecting the change in absorbance at 240 nm due to the presence of H_2_O_2_. One unit of CAT activity was defined as the amount of crude enzyme needed to catalyze 1 μmol of H_2_O_2_ per minute.

For polyphenol oxidase (PPO) activity determination, 100 μL of the supernatant was added to 1 mL of reaction buffer containing 100 mM PBS (pH 6.8) and 50 mM catechol, and the absorbance change at 420 nm was monitored. One unit of PPO was defined as 1 nmol of catechol oxidation per minute. For the above determination, each group contained three replicates, and each determination was repeated 3 times.

### 2.8. Statistical Analysis

The experiment was conducted with three replicates, and the data was presented as means ± SD. Statistical analysis was performed using one-way analysis of variance. The Duncan test was chosen for the significant difference analysis conducted using SPSS version 23.0 (IBM Co., New York, NY, USA), and OriginPro 9.0 (Origin Lab Co., Northampton, MA, USA) was used for graphical representation.

## 3. Results

### 3.1. Validation and Pathogenicity of P. expansum

**Figure 1:**
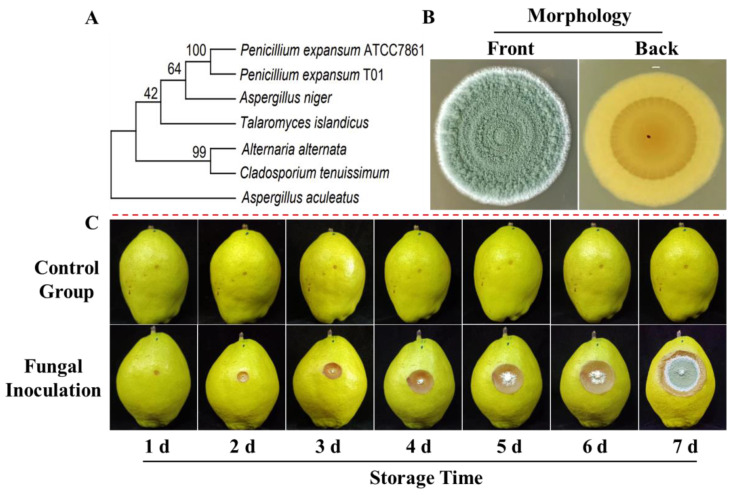
Validation and pathogenicity of *P. expansum* T01: (**A**) phylogenetic tree of *P. expansum* T01; (**B**) morphology of *P. expansum* T01; and (**C**) pathogenicity and infection of *P. expansum* T01 to pears.

*P. expansum* was verified through ITS sequencing, and the phylogenetic tree indicated that *P. expansum* T01 shared significant similarity with *P. expansum* ATCC7861 (Figure 1A). Morphological observations revealed that the colony’s front color was grass green and the reverse was dark yellow, consistent with the typical morphology of *P. expansum* (Figure 1B). Pathogenicity analysis demonstrated that *P. expansum* T01 was capable of infecting pears, causing blue-mold rot. The lesions on the pears expanded over time, with hyphae appearing on the rot surface during the later stages of storage (Figure 1C).

### 3.2. MIC and MFC of Oregano Essential Oil Against P. expansum

The antifungal activity of OEO against *P. expansum* was evaluated by determining the MIC and MFC, as shown in Table 1. After 48 h of incubation under aerobic conditions, no spore growth was observed in the PDB medium supplemented with 0.02% OEO or at higher concentrations. However, a concentration of 0.01% OEO did not completely inhibit spore growth. The MIC of OEO against *P. expansum* was thus determined to be 0.02%. Further incubation for another 48 h at 30 °C revealed spore growth in the group supplemented with 0.02% OEO, but not in groups treated with 0.05% or higher concentrations, suggesting spore viability was compromised. Consequently, the MFC was established as 0.05%.

### 3.3. Spore Germination of P. expansum in Response to Oregano Essential Oil

**Figure 2:**
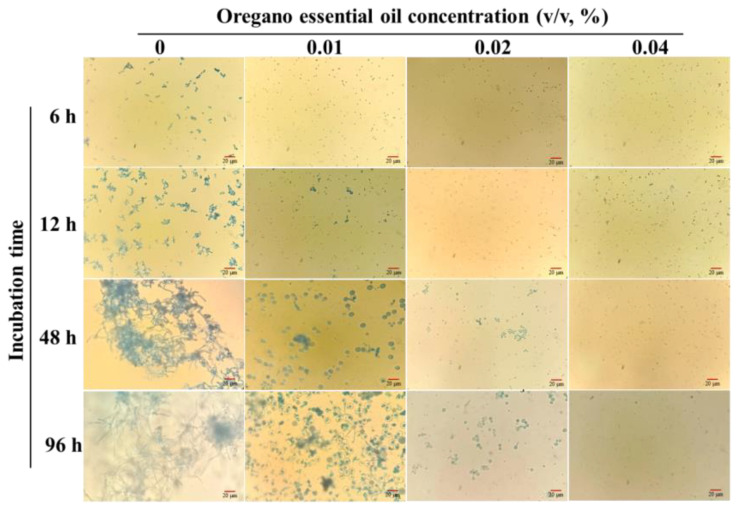
Spore germination of *P. expansum* against oregano essential oil.

The impacts of various concentrations of OEO on the spore germination of *P. expansum* are depicted in Figure 2. Over time, the spores in the control group swelled, with a germination rate of approximately 34.18% recorded after 12 h, while no germination occurred in the OEO-treated groups. Spores exposed to 0.01% OEO began to germinate after 48 h, with a low germination rate of 3.33%, even after 96 h. At concentrations of 0.02% or higher, no spore germination was observed throughout the 48 h culture period. Rare germination occurred in the group supplemented with 0.02% OEO after 96 h, while no germination was observed with the 0.04% treatment. These results indicated that OEO inhibited spore germination in a dose-dependent manner. Spore morphology and size remained unchanged at higher concentrations, likely due to the fungicidal effects of OEO, functioning by disrupting the normal physiological metabolism.

### 3.4. Hyphae Development of P. expansum in Response to Oregano Essential Oil

**Figure 3:**
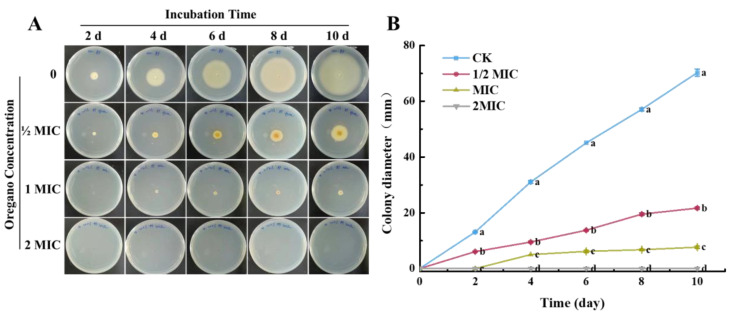
Effect of oregano essential oil on hyphae development of *P. expansum*: (**A**) hyphae development of *P. expansum* under the treatment of oregano essential oil at different concentrations; (**B**) colony diameter of *P. expansum* with various concentrations of oregano essential oil. The CK represents the control group, without any addition of oregano essential oil. The data are presented as means ± SD. The different letters indicate significant differences based on Duncan’s test (*p* < 0.05).

Hyphae development in the presence of OEO is illustrated in Figure 3. Hyphae continued to expand with increased storage time. However, growth was significantly inhibited by OEO, as evidenced by reduced diameters of colonies compared to the control group (Figure 3A). Specifically, the colony diameter in the control group showed a linear increase, reaching 70.17 mm by the 10th day. In contrast, diameters were only 21.67 mm and 7.67 mm in the presence of 1/2 MIC and MIC concentrations, respectively, representing decreases of 69.1% and 89.1% (Figure 3B). Furthermore, no mycelium growth was observed until the 4th day at the MIC concentration, and growth was completely inhibited at 2 MIC, indicating a viability loss or the unculturable state of the spores under such conditions.

### 3.5. Plasma Membrane Integrity of P. expansum in Response to Oregano Essential Oil

**Figure 4:**
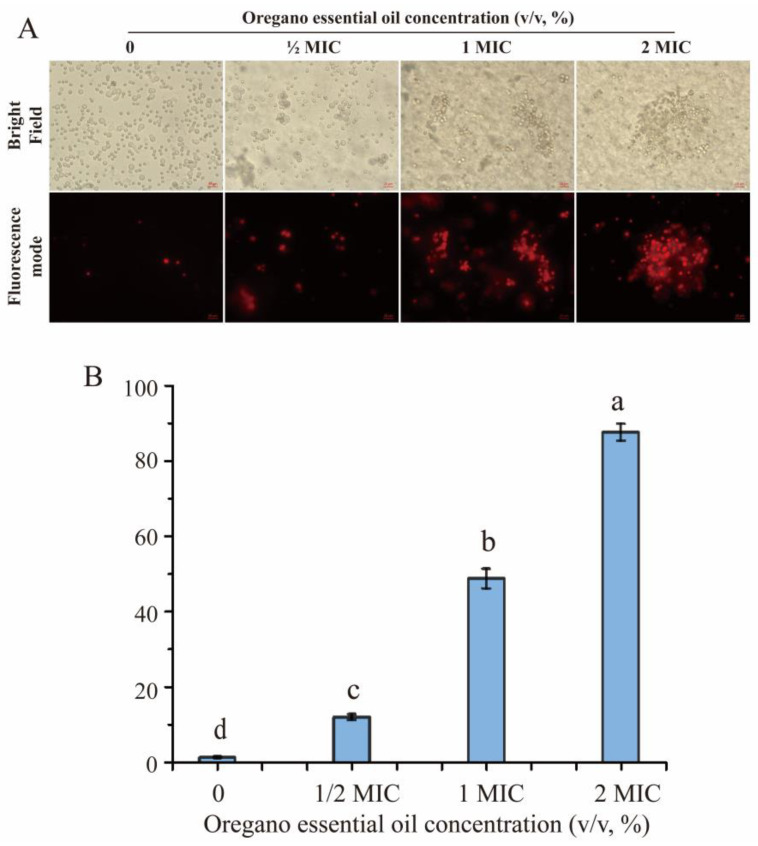
PI staining of *P. expansum* spores with various concentrations of oregano essential oil: (**A**) spore morphology with PI staining under a bright field and using fluorescent mode; (**B**) PI staining rate of spores under the treatments of various concentrations of oregano essential oil. The data are presented as means ± SD. The different letters indicate significant differences based on Duncan’s test (*p* < 0.05).

Plasma membrane integrity of spores was assessed using PI staining, and the stained spores under bright field and fluorescent mode are shown in Figure 4. Spores in the control group without OEO supplementation swelled and germinated after 12 h, while those under MIC and 2 MIC concentrations were smaller and ungerminated. This suggested that OEO disrupted spore development by affecting the germination process. In fluorescent mode, the number of stained cells increased with higher concentrations of OEO. The PI staining rate at 2 MIC was 87.59%, representing a 7.38-fold increase compared to 1/2 MIC. These results indicated that OEO damaged plasma membrane integrity in a concentration-dependent manner, leading to changes in membrane permeability and disruption of substance exchange, thus inhibiting spore germination. 

### 3.6. Leakage of Intracellular Components of P. expansum in Response to Organo Essential Oil

**Figure 5:**
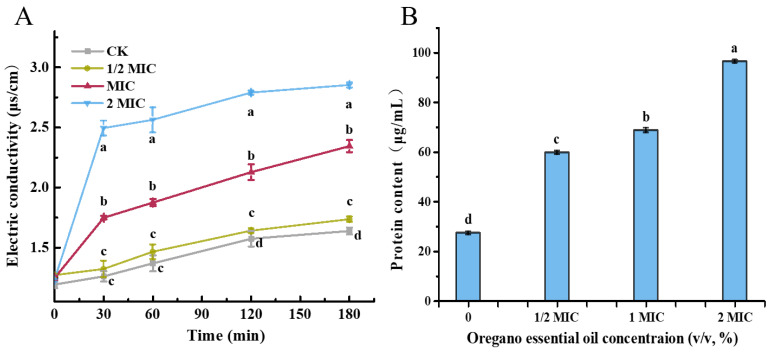
Leakage of intracellular components of *P. expansum* in response to oregano essential oil. (**A**) Extracellular electric conductivity of *P. expansum* exposed to oregano essential oil. The CK represents the control group, without exposure to oregano essential oil. (**B**) Extracellular protein content of *P. expansum* under the treatment of oregano essential oil. The data are presented as means ± SD. The different letters indicate significant differences based on Duncan’s test (*p* < 0.05).

Changes in plasma membrane permeability led to the leakage of intracellular components, as indicated by increased extracellular electric conductivity shown in Figure 5A. Conductivity rose gradually over time in both treatment and control groups, with higher concentrations of OEO leading to greater increases. After 30 min, the conductivity values for MIC and 2 MIC treatment groups were 1.88 μs/cm and 2.56 μs/cm, respectively, representing increases of 37.2% and 86.9% compared to the control group (1.37 μs/cm). As depicted in Figure 5B, the extracellular protein content increased with higher concentrations of OEO, indicating a steady release of intracellular proteins. The protein content under the treatments of 1 MIC and 2 MIC concentrations of OEO was 68.74 and 96.44 μg/g mycelium, respectively, representing increases of 151.18% and 252.36% compared with the control group (27.37 μg/mg). The steady elevation of electric conductivity and leakage of intracellular proteins suggested membrane damage as one of the mechanisms by which OEO inhibited fungal growth. 

### 3.7. Incidence of Pear Blue Mold in Response to Oregano Essential Oil

**Figure 6:**
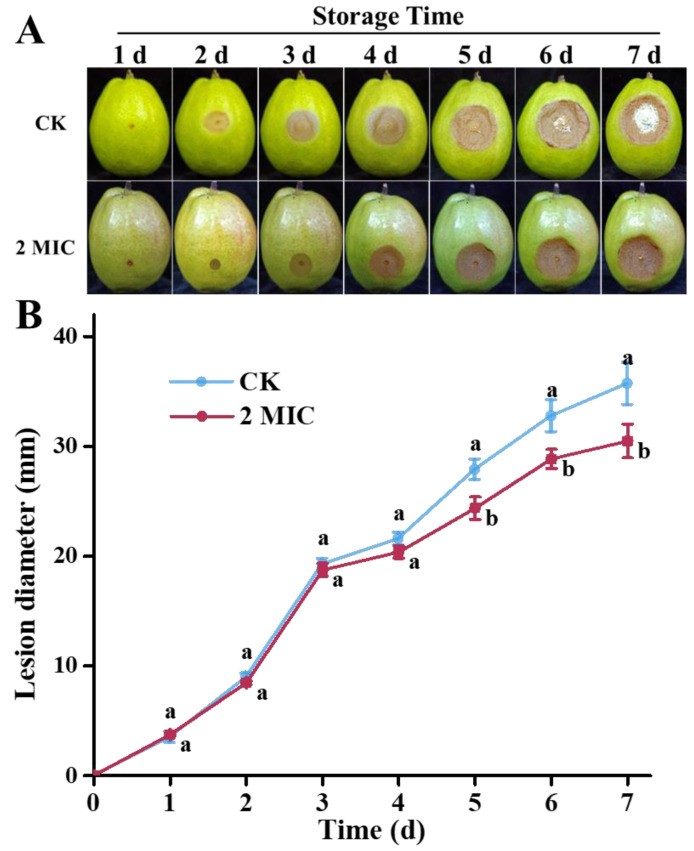
Development of blue-mold rot in response to oregano essential oil: (**A**) lesion development in pears upon the treatment with oregano essential oil and (**B**) lesion diameters of pears under the treatment of oregano essential oil. The CK represents the control group, without oregano oil treatment. The data are presented as means ± SD. The different letters indicate significant differences based on Duncan’s test (*p* < 0.05).

To evaluate the effectiveness of OEO against blue-mold rot of pears, a chitosan-loaded oregano essential oil film was applied to the fruit. The development of blue mold is depicted in Figure 6. With increased storage time, the progression of blue mold was inhibited by the OEO treatment, and the lesion expansion on pear fruit was reduced by 12.56%, 11.83%, and 14.54% on the 5th, 6th, and 7th day, respectively. Under normal storage conditions, spores and hyphae could easily spread in the air, potentially infecting other fruits. Notably, hyphae of *P. expansum* appeared on the rot surface after 6 days of storage but continued to expand, although no extrinsic hyphae were observed throughout the storage period. These results demonstrated that the oregano–chitosan film showed a significant inhibitory effect on lesion expansion and blue-mold development, thus reducing the risk of spread of *P. expansum*.

### 3.8. Color Change of Pear Fruit in Response to Oregano Essential Oil

Color changes in pears treated with or without OEO are presented in Table 2. Over the 15-day storage period, there were no significant differences between the treatment and control groups in terms of L* and b* values (*p* > 0.05). While there were some fluctuations in the a* value during the later stages of storage, there were no noticeable color differences overall, indicating that OEO treatment did not negatively affect pear color during storage.

### 3.9. Firmness, Titratable Acidity, and Soluble-Solid Content of Pears Treated with Oregano Essential Oil

**Figure 7:**
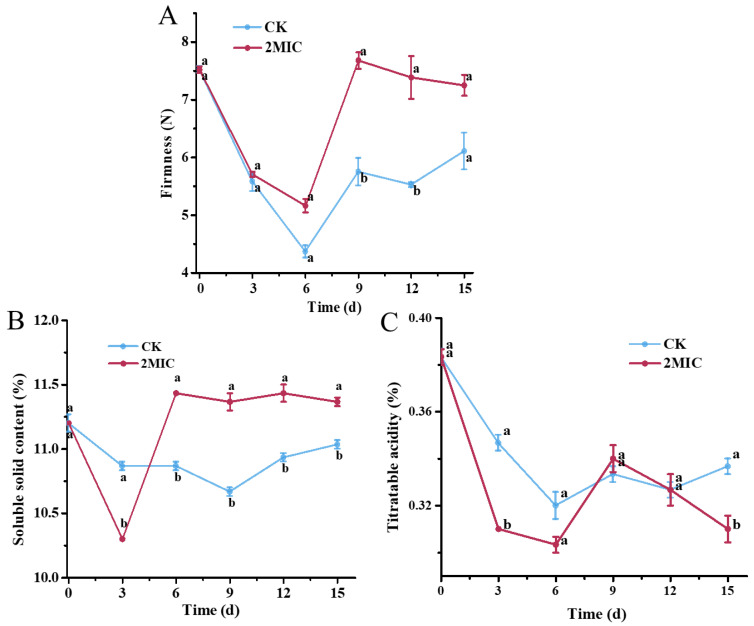
The quality attributes of pear fruit in response to oregano essential oil during storage: (**A**) firmness of pears under the treatment of oregano essential oil; (**B**) soluble-solid content of pears under the treatment of oregano essential oil; and (**C**) titratable acidity of pears under the treatment of oregano essential oil. The CK represents the control group, without oregano oil treatment. The data are presented as means ± SD. The different letters indicate significant differences based on Duncan’s test (*p* < 0.05).

Firmness is an indicator of fruit maturity. As storage time increased, the firmness of pears treated with OEO remained high, with significant differences observed on the 9th and 12th day. Specifically, firmness values for the treated group on the 9th, 12th, and 15th day were 7.68 N, 7.39 N, and 7.25 N, respectively, representing increases of 33.57%, 33.66%, and 18.72% compared to the control group (Figure 7A). The soluble-solid content exhibited a similar trend, with the OEO treatment helping to inhibit the decline in soluble-solid content and maintain higher levels throughout the storage period. The soluble-solid content in the OEO treatment group was 3% higher than that in control group on the 15th day (Figure 7B). Throughout the storage period, titratable acidity demonstrated fluctuating trends of decrease and increase. The OEO treatment reduced the titratable acidity. Specifically, the total titratable acidity of the treated group was 7.9% lower than that in the control group on 15th day (Figure 7C).

### 3.10. Water Loss and Respiration Rate of Pear Fruits Treated with Oregano Essential Oil

**Figure 8:**
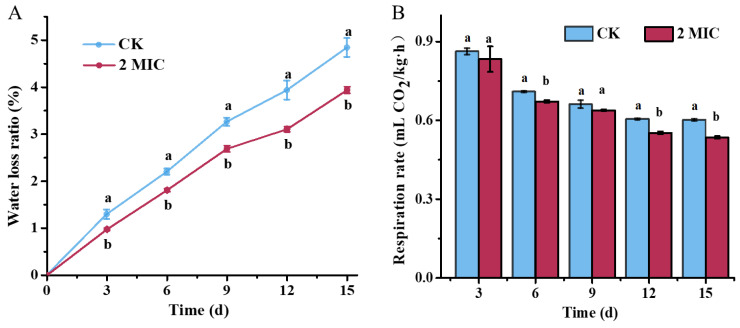
Water loss and respiration rate of pear fruits against oregano essential oil: (**A**) water loss of pears in response to oregano essential oil treatment and (**B**) respiration rate of pears in response to oregano essential oil treatment. The CK represents the control group, without oregano oil treatment. The data are presented as means ± SD. The different letters indicate significant differences based on Duncan’s test (*p* < 0.05).

Water loss reflects the freshness of fruits and vegetables. As shown in Figure 8, water loss increased with extended storage time, but was reduced in the OEO treatment group, compared to the control group. Over the entire 15-day storage period, the total water-loss rate for the control group was 4.84%, while it was 3.94% for the treated group, representing a decrease of 18.6% (Figure 8A). As for respiration rate, all groups exhibited a similar declining trend over 15 days of storage. The OEO treatment reduced the respiration rate by 10.92% compared to that in control group (0.6 mL CO_2_/kg/h) on 15th day (Figure 8B). This reduction in respiration rate was conducive to decreased carbohydrate consumption and nutrient metabolism, thereby helping to maintain pear quality.

### 3.11. Enzymatic Activity of Pear Fruits Treated with Oregano Essential Oil

**Figure 9:**
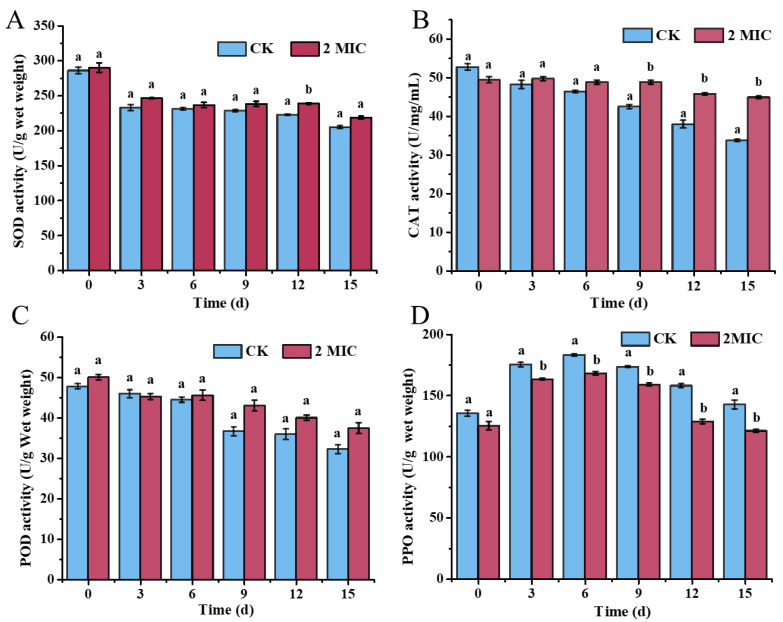
Enzymatic activity of pear fruits in response to oregano essential oil: (**A**) SOD activity of pears in response to oregano essential oil treatment; (**B**) CAT activity of pears in response to oregano essential oil treatment; (**C**) POD activity of pears in response to oregano essential oil treatment; and (**D**) PPO activity of pears in response to oregano essential oil treatment. The CK represents the control group, without oregano oil treatment. The data are presented as means ± SD. The different letters indicate significant differences based on Duncan’s test (*p* < 0.05).

The antioxidant enzyme system composed of SOD, CAT, and POD is crucial for maintaining redox balance. As depicted in Figure 9A, SOD activity in both control and treated groups decreased with prolonged storage. However, the OEO treatment mitigated the decline in SOD activity. The SOD activity in the treated pears was 218.53 U/g on the 15th day, which was 6.69% higher than that in the control group (Figure 9A).

CAT activity followed a similar decreasing trend throughout the storage period. The OEO treatment slowed this decline, resulting in significantly higher CAT activity after 15 days compared to the control group, with the latter showing a 24.83% reduction (*p* < 0.05) (Figure 9B). Although no significant difference was observed in POD activity, the OEO treatment lessened its decline over time. Compared with the initial values, POD activity decreased by 32.56% in the control group and 25.19% in the treated group, suggesting that OEO helped to maintain POD activity during storage, thus reducing the elevation in reactive oxygen species (Figure 9C).

PPO was involved in the fruit browning process, and the PPO activity showed a trend of initial increase followed by a decrease over 15 days of storage, with peak activity on the 6th day (Figure 9D). The OEO treatment reduced the peak PPO activity on the 6th day to 168 U/g, which was 8.252% lower than in the control group. By the 15th day, a significant difference was observed, representing a 14.98% decrease in PPO activity in the treated group compared to the control group. These results suggested that OEO enhanced the activation of the antioxidant system and reduced ROS generation during postharvest storage, thereby delaying the quality deterioration of fruits.

## 4. Discussion

The rising demand for natural alternatives to synthetic fungicides has stimulated the exploration of essential oils as to their use in postharvest management. In this study, the OEO exhibited notable inhibitory effects, with determined MIC (0.02%) and MFC (0.04%) against *P. expansum* that highlighted its potential as a viable alternative to synthetic fungicides. Many research efforts have documented the antifungal properties of essential oils against postharvest pathogens. Sun et al. [7] established that the MIC and MBC of *Litsea cubeba* essential oil against *Penicillium digitatum* were 1 μL/mL and 8 μL/mL, respectively. Buonsenso et al. [14] reported that essential oils derived from basil at concentrations of 1.0% and 0.5% effectively inhibited the mycelial growth of *P. expansum*. In light of these findings, the present study not only demonstrated the superior antibacterial activity of OEO relative to that of many other natural compounds documented in the literature, but also distinguished itself by focusing specifically on the efficacy of OEO in *Pyrus sinkiangensis* pears, a fruit that has not been extensively studied in this context.

The plasma membrane plays a crucial role in maintaining cellular homeostasis, facilitating material exchange, and ensuring normal cellular function [31]. In the present study, treatment with OEO significantly altered the permeability and integrity of the plasma membrane in *P. expansum*, as evidenced by the results from PI staining, increased protein release, and elevated electric conductivity measurements. These findings suggested that OEO compromised the structural integrity of the fungal cells, thus leading to impaired functionality. Similarly, Dutra et al. [32] demonstrated that exposure to OEO resulted in cell wall destruction and deformation of cellular integrity in *Alicyclobacillus* spp. This parallel underscores the broader antifungal potential of OEO across different fungal species. The antifungal mechanism of OEO can likely be attributed to its ability to disrupt the fungal cell membrane, which not only leads to leakage of intracellular components but also ultimately results in cell death [15].

In addition to its antifungal properties, OEO treatment demonstrated significant reductions in weight loss, maintenance of firmness, and preservation of SSC in the treated fruits. These findings highlighted the dual role of OEO, functioning not only as an antifungal agent but also as a quality enhancer during storage. The observed reduction in weight loss was particularly noteworthy, as it suggested that OEO formed a protective barrier on the fruit surface, thereby minimizing moisture evaporation. This is consistent with the findings of Promwee and Matan [33], who reported that essential oils can create a semi-permeable film that effectively regulates gas exchange and moisture loss, contributing to the overall quality of stored fruits. Firmness is a key quality parameter that directly influences consumer acceptance and marketability. Our data indicated that OEO treatment effectively preserved the mechanical integrity of pears, which was essential for their storage and transportation. The retention of firmness can be attributed to the oil’s ability to modulate the activity of cell-wall-degrading enzymes responsible for fruit softening during ripening. This finding aligns with previous studies that have demonstrated the inhibitory effects of essential oils on such enzymes [34].

During the ripening process, fruits undergo physiological changes that increase their susceptibility to oxidative stress, thereby triggering various physiological deterioration pathways. Antioxidant enzymes, primarily composed of SOD, CAT, and POD, play a crucial role in scavenging reactive oxygen species generated during these metabolic processes, thus preventing oxidative damage to cellular components [35]. SOD catalyzes the dismutation of superoxide radicals into hydrogen peroxide and molecular oxygen, and its activity and concentration serve as indicators of ripeness and senescence in fresh produce [36]. Our results demonstrated that pears treated with OEO exhibited a 6.69% higher SOD activity compared to the control group after 15 days of storage. This enhancement corroborates the findings of Taheri et al. [37], who reported that natural plant extracts significantly elevated SOD activity in treated pepper. Additionally, as illustrated in Figure 9B, OEO-treated pears maintained a 24.83% higher CAT activity compared to the control group after the same storage period. The elevated CAT activity suggested that OEO effectively mitigated the accumulation of hydrogen peroxide and subsequent oxidative damage. Shao et al. [38] also demonstrated that tea tree oil vapor treatment can enhance CAT activity, thereby improving the overall antioxidant capacity of postharvest strawberry. The activation of both SOD and CAT indicated that OEO may stimulate the intrinsic antioxidant mechanisms of pears, which is essential for maintaining cellular integrity during postharvest storage [39].

PPO is involved in the enzymatic browning process; our findings revealed a slight reduction in PPO activity in treated pears during storage, although this decrease was less pronounced than that observed in the control group. This observation may be attributed to the inhibitory effects of specific bioactive compounds present in OEO in PPO activity. Previous studies have shown that certain components of essential oils can effectively inhibit PPO, thereby reducing enzymatic browning and preserving fruit quality [40,41]. The implications of these findings are significant, as they highlight the multifaceted role of OEO in enhancing the antioxidant defense system of pears. By improving the activities of SOD and CAT, OEO not only helps combat oxidative stress but also plays a crucial role in maintaining the sensory and nutritional attributes of fruit during storage [42]. This positions OEO as a promising candidate for natural postharvest management strategies aimed at prolonging shelf life and enhancing the marketability of fruits.

As consumer demand for organic and chemical-free produce increases, integrating essential oils into postharvest practices offers a sustainable alternative to synthetic fungicides [43]. Understanding the interaction between essential oils and fruit physiology is crucial for optimizing their application in food preservation. While previous research has predominantly focused on the antifungal properties of essential oils, our study specifically examines the sensory implications of OEO treatment on pears. Essential oils consist of various volatile compounds that can significantly influence sensory attributes such as taste and aroma [44]. Future research should prioritize the impact of OEO on the sensory qualities of pears to ensure consumer acceptance, especially given the rising preference for natural products. In addition, challenges still remain in the development and utilization of essential oils, such as poor water solubility, volatility, and instability; these can limit the effectiveness and consumer appeal of these oils [45]. Additionally, exploring the synergistic effects of essential oils with other preservation methods, such as refrigeration and modified atmosphere packaging, could enhance their overall efficacy. While our findings provide a strong foundation for further exploration of OEO as a natural preservative, there are limitations to our study that warrant consideration. Specifically, the long-term effects of OEO on various fruit types and under different storage conditions remain to be fully elucidated.

## 5. Conclusions

In postharvest management, it is critical to effectively control postharvest decay without compromising the quality attributes of fruits. In this study, a comprehensive evaluation of OEO’s effects on both antifungal activity and quality attributes of *Pyrus sinkiangensis* fruit was conducted. The OEO not only inhibited the growth of *P. expansum* but also enhanced the overall quality of the fruit during storage. OEO was found to disrupt *P. expansum* by damaging the integrity of the fungal plasma membrane, as evidenced by changes in plasma membrane permeability and the leakage of cellular components. The OEO treatment significantly reduced weight loss, maintained firmness, and preserved soluble-solid content in the treated pears. In addition, OEO treatment stimulated the intrinsic antioxidant mechanism and mitigated damage caused by reactive oxygen species. This study provides compelling evidence for the antifungal and quality-preserving properties of OEO in the postharvest management of pears, underscoring its potential as an alternative to synthetic fungicides for controlling these aspects of storage management. This study not only expands the application scope of OEO but also offers a theoretical foundation for future studies and practical applications in postharvest management. Future research should focus on elucidating the detailed mechanisms of action, exploring the sensory implications, and optimizing application methods to maximize the benefits of OEO in postharvest technology. Evaluating the anti-*P. expansum* activity of pure thymol and carvacrol is also essential for helping us gain a comprehensive understanding of their antimicrobial mechanisms.

## Figures and Tables

**Table 1:** MIC and MFC of oregano essential oil against *P. expansum*.

Incubation Time		Concentration of Oregano Essential Oil (%, *v*/*v*)					
		0	0.01	0.02	0.04	0.05	0.1
48 h	Parallel 1	+++	+	−	−	−	−
	Parallel 2	+++	+	−	−	−	−
	Parallel 3	+++	+	−	−	−	−
96 h	Parallel 1	+++	++	+	+	−	−
	Parallel 2	+++	++	+	+	−	−
	Parallel 3	+++	++	+	+	−	−

Note: (−) represents no growth; (+) represents growth.

**Table 2:** Color change of pear fruit during a storage period of 15 days.

Treatment Group	Parameters	Storage Time					
		0 Days	3 Days	6 Days	9 Days	12 Days	15 Days
CK	L*	45.5 ± 2.13 ^a^	44.1 ± 1.16 ^a^	48.3 ± 0.49 ^a^	42.9 ± 0.05 ^a^	45.7 ± 0.58 ^a^	47.1 ± 0.26 ^a^
	a*	−6.5 ± 0.26 ^a^	−6.5 ± 0.42 ^a^	−6.5 ± 0.22 ^a^	−5.6 ± 0.17 ^a^	−5.7 ± 0.22 ^a^	−5.4 ± 0.19 ^a^
	b*	33.9 ± 0.60 ^a^	33.6 ± 1.35 ^a^	36.5 ± 0.76 ^a^	31.6 ± 0.85 ^a^	31.4 ± 0.49 ^a^	32.6 ± 1.70 ^a^
2 MIC	L*	45.8 ± 0.38 ^a^	43.6 ± 3.33 ^a^	39.7 ± 0.82 ^b^	43.0 ± 0.15 ^a^	45.5 ± 0.57 ^a^	47.6 ± 0.4 ^a^
	a*	−7.1 ± 0.08 ^a^	−7.3 ± 0.27 ^a^	−7.2 ± 0.16 ^a^	−6.3 ± 0.34 ^a^	−7.1 ± 0.32 ^b^	−6.8 ± 0.16 ^b^
	b*	32.2 ± 0.33 ^a^	33.4 ± 1.98 ^a^	34.4 ± 0.52 ^a^	34.6 ± 1.08 ^a^	34.7 ± 2.18 ^a^	37.3 ± 0.40 ^a^

Note: L*: lightness; a*: redness; b*: yellowness. Values are means ± standard deviations. Means represented by different lowercase letters in the same row are significantly different (*p* < 0.05).

## Data Availability

All data are contained within this article.
